# High-Frame-Rate Doppler Ultrasound Using a Repeated Transmit Sequence

**DOI:** 10.3390/app8020227

**Published:** 2018-02-01

**Authors:** Anthony S. Podkowa, Michael L. Oelze, Jeffrey A. Ketterling

**Affiliations:** 1 Beckman Institute, University of Illinois at Urbana-Champaign, Urbana, IL 61801, USA; 2 Lizzi Center for Biomedical Engineering, Riverside Research Institute, New York, NY 10038, USA

**Keywords:** color flow doppler, high-frequency ultrasound, plane-wave imaging, Nyquist velocity, multirate signal processing

## Abstract

The maximum detectable velocity of high-frame-rate color flow Doppler ultrasound is limited by the imaging frame rate when using coherent compounding techniques. Traditionally, high quality ultrasonic images are produced at a high frame rate via coherent compounding of steered plane wave reconstructions. However, this compounding operation results in an effective downsampling of the slow-time signal, thereby artificially reducing the frame rate. To alleviate this effect, a new transmit sequence is introduced where each transmit angle is repeated in succession. This transmit sequence allows for direct comparison between low resolution, pre-compounded frames at a short time interval in ways that are resistent to sidelobe motion. Use of this transmit sequence increases the maximum detectable velocity by a scale factor of the transmit sequence length. The performance of this new transmit sequence was evaluated using a rotating cylindrical phantom and compared with traditional methods using a 15-MHz linear array transducer. Axial velocity estimates were recorded for a range of *±*300 mm/s and compared to the known ground truth. Using these new techniques, the root mean square error was reduced from over 400 mm/s to below 50 mm/s in the high-velocity regime compared to traditional techniques. The standard deviation of the velocity estimate in the same velocity range was reduced from 250 mm/s to 30 mm/s. This result demonstrates the viability of the repeated transmit sequence methods in detecting and quantifying high-velocity flow.

## 1. Introduction

Doppler ultrasound is widely used to estimate velocity of blood flow and tissue motion in biological specimens [1]. Traditional Doppler approaches estimate the axial component of motion, where the maximum velocity is limited by the pulse-repetition frequency (PRF) of the ultrasound transmission as described by the Doppler equation (see Equation (9) in Section 2.1.5). In the most common estimation algorithms, such as the lag-one autocorrelation approach [2], velocities that exceed this limit result in phase wrapping (i.e., aliasing) and can result in estimates in the opposite direction of the actual flow. However, using conventional ultrasonic methods, if the Doppler estimation occurs throughout an image frame, such as in color flow Doppler, then the maximum velocity is now limited by the frame rate.

With conventional ultrasonic methods, images are formed by using one focused transmit per lateral scan line. This design decision, while simple to implement and well understood, results in a temporal bottleneck on the frame rate. Because the total acquisition time for each transmit event is the round-trip time of flight to the maximum depth of interest and back, the total image acquisition time is scaled by the number of transmit events (on the order of 128 or more). Thus, if high frame rate images are desired, reducing the number of transmit events for each frame is paramount.

Coherent compounding approaches were introduced to minimize the number of transmit events for each image frame and thereby increase the absolute frame rate without sacrificing image quality. These approaches include limited diffraction beams [3–5], steered plane waves [6], and diverging waves [7,8]. Each of these methods produces high-quality images at frame rates in the kHz range. Acquiring data at the highest frame rate is paramount for accurate reconstruction of high velocity motion, such as that observed in mouse blood flow, where velocities can be as high as 900 mm/s [9]. This is particularly true for high-frequency ultrasound (>20 MHz) because, based on the Doppler equation, an increase in transmit frequency will lower the maximum velocity that can be resolved before aliasing.

Compounding approaches result in an effective slow-time downsampling of the data, thus reducing the frame rate by a factor of *N_ϕ_*, where *N_ϕ_* is the number of angles transmitted per compounded frame. In addition, because the plane-wave compounding approach relies on a stationary medium assumption, the compounding process can result in signal loss when particles move a half-integer multiple of a wavelength over the compounding interval [10]. For this reason, using the precompounded data is advantageous for high velocity imaging. This idea has been successfully exploited to compensate for motion artifacts in coherent compounding techniques [11,12].

Use of the precompounded data for Doppler estimation comes with its own costs. Because each precompounded image is transmitted at a different steering angle, sidelobes in the point spread function (PSF) rotate about the target location even when the target is stationary. This sidelobe motion results in a periodic noise source which can corrupt velocity estimates. In order to address this issue, we propose using a repeated transmission sequence to avoid sidelobe motion on a short time scale. Specifically, we propose two new approaches which use this new transmit sequence. The first method estimates flow by evaluating the slow-time autocorrelation between adjacent precompounded images at the same transmit steering angle and aggregating the individual autocorrelation estimates over all angles. The second approach generates compounded frames for the initial and repeat events, respectively, and correlates these two frames. For both approaches, the autocorrelation occurs at a lag equivalent to one transmit event as opposed to a full compounded frame, resulting in an increase of the Nyquist velocity on the order of the number of angles used in the transmit sequence.

The proposed techniques were validated by using a cylindrical hyperechoic phantom mounted on a motor rotated at a known angular velocity. Data were acquired using two traditional linear transmit sequences of different lengths as well as the new double transmit sequence. For each acquired dataset, the received in-phase and quadrature (IQ) data were beamformed, and velocities were estimated using both the proposed and traditional methods for both the compounded and precompounded datasets. Power Doppler and color flow Doppler images were generated, and the root-mean-square error, standard deviation, and bias were evaluated for each processing scheme.

## 2. Materials and Methods

### 2.1. Theory

#### 2.1.1. Coherent Plane-Wave Compounding

**Figure 1:**
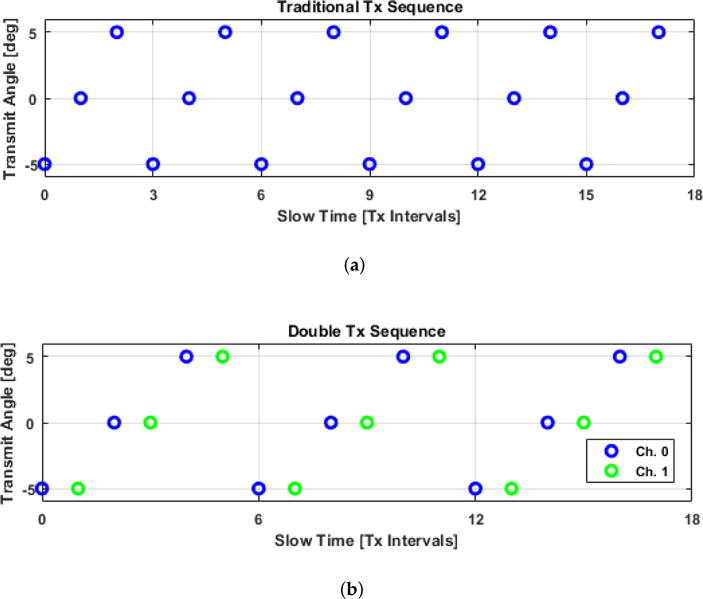
Comparison of the different transmit sequences used in this study. With the traditional transmit sequence, acquisitions with the same point spread function are spaced apart over intervals equivalent to a transmit sequence length. Due to the fact that these components will exhibit the strongest spatial correlations, reordering them such that they are temporally adjacent allows for correlation estimates at the shortest possible time interval. (**a**) Traditional transmit sequence where the transmit angles linearly increment and then repeat; (**b**) Double transmit sequence where the transmit angle repeats before linearly incrementing to the next angle.

A plane-wave transmission is generated by firing all of the elements of a linear array with inter-element transmission delays that create a coherent wave front at a steered angle. The return echoes are then digitized on all of the array elements and beamformed data representing a full image frame can be obtained with delay-and-sum methods. Plane waves are usually transmitted in a sequence of *N_T_* steered excitations spanning a set of *N_ϕ_* distinct angles, where the angle of transmission linearly increases from low to high (Figure 1a). Note that in the traditional case *N_T_* = *N_ϕ_*. The data collected from each transmit event, after beamforming, are coherently summed to form high-resolution images (HRIs), which improves image resolution and contrast [6].

The fundamental tradeoff when using a coherent compounding technique is between the spatial and temporal resolution. As noted in [13], the effect of the coherent compounding process is one of a temporal low-pass, finite impulse response (FIR) filter. However, Ekroll, et al. [13], did not address the effect of the downsampling operation. As described by multirate signal processing theory [14,15], a downsampling operation results in an effective expansion of the discrete time spectrum, which can result in additional aliasing. Because traditional coherent compounding techniques use uniform weights [6], the anti-aliasing filter corresponds to a rectangular filter (See Appendix) that is prone to temporal frequency sidelobes. Equally weighting the compounded components could lead to slow-time temporal aliasing and high velocity estimates would be particularly vulnerable to this source of error.

In principle, one could design an anti-aliasing filter with better stop band characteristics using traditional signal processing techniques [15]. However, such a design process would inevitably produce a nonuniformly weighted low pass filter, and as such would result in reduced signal contribution from the highly steered components of the compounding process. Consequently, the spatial resolution of the compounded images would be reduced.

**Figure 2:**
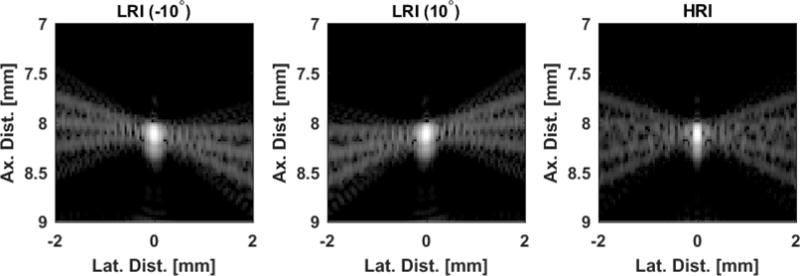
The coherent compounding approach generates high-resolution images (HRIs)by summing together low-resolution images (LRIs) formed at different steering angles. If we look at each LRI individually, we can see that the point spread function (PSF) rotates about the center as the steering angle changes. If we use the LRIs to produce Doppler estimates, the sidelobe rotation could be misinterpreted as tissue motion by the lag-one autocorrelation algorithm. Images are rendered at a 60-dB scale and are normalized to their corresponding maxima.

Another approach would be to use precompounded low-resolution images (LRIs). However, because each of the steered plane waves produces a slightly different PSF, a new temporal noise source is introduced in the form of spatial sidelobe motion that oscillates periodically in slow time (Figure 2). This sidelobe motion could be mistaken as tissue motion by traditional Doppler estimation techniques [2], especially in hypoechoic tissue regions. While in principle such sidelobes could be minimized via an appropriately designed apodization function, such a design choice ultimately results in a loss of spatial resolution. As such, it is important to consider alternative processing schemes to address this new noise source associated with LRI-based Doppler estimation.

#### 2.1.2. The Polyphase Decomposition

A potential alternative to resolve this issue is to decompose the slow-time signal into its *N_T_* polyphase components [14]. In the field of multirate signal processing, an *N*th order polyphase decomposition of a discrete signal *s*[*n*] is a set of *N* periodically interleaved subsequences sampled at 1/*N* times the framerate. Mathematically, the *k*th polyphase component signal *e_k_*[*n*] is given by

(1) $$e_{k}[n]=s[Nn+k]$$

(2) k∈{0:N−1}.

If we preserve all the polyphase components, the original signal can be reconstructed exactly by interleaving, such that

(3) $$s[n]=e_{\mathrm{mod}(n,N)}\left[\left\lfloor \frac{n}{N}\right\rfloor \right],$$

where mod(*n*, *N*) is the remainder after division of *n* by *N* and ⌊·⌋ is the floor function.

Noting that if we choose the polyphase decomposition order *N* such that *N* = *N_T_*, each polyphase component in the slow-time signal corresponds to a particular angle in the transmit sequence. Direct comparisons can be made between slow-time signals of the same transmit steering angle, and thus would be insensitive to sidelobe motion. However, if we choose to do so with a conventional linear sequence, there is no performance improvement relative to just using the HRIs, as each polyphase channel operates at the HRI frame rate. For this reason, we introduce the double transmit sequence as a workaround, as examined in the next subsection.

#### 2.1.3. Double Transmit Sequence

In order to mitigate the effect of sidelobe motion in the precompounded LRIs, a repeated transmit sequence is proposed (Figure 1b). By repeating the transmit angle each time, motion can be analyzed between frames with the same PSF at a shorter time interval than would be possible with a traditional linearly incrementing transmit sequence. Such an approach opens up the design space to new Doppler estimation approaches (Sections 2.1.6 and 2.1.7).

One consequence of this design decision is that it doubles the length of the transmit sequence such that *N_T_* = 2*N_ϕ_* instead of *N_ϕ_* as before. This means that if we choose to compound the sequence to generate HRIs for review, the HRI frame rate will be half that of the linear transmit sequence. However, given that the maximum velocity range is increased by a factor of *N_ϕ_* as described in Sections 2.1.6 and 2.1.7, and that typically one averages over multiple HRI frames anyway, one can argue that the decision is justifiable. That being said, the efficacy of such an approach should be evaluated by the end user to determine if tradeoff is acceptable.

#### 2.1.4. Clutter Filtering

Prior to estimating velocity, a clutter filter is typically used to suppress stationary components of the slow-time signal. Traditionally this is accomplished with a high-pass filter. In the case of HRIs, the PSF of each frame is the same and, therefore, clutter filtering may be handled with standard digital filtering techniques [15]. However, because the PSF in each LRI frame varies with time, the options for clutter filtering become more varied. If we wish to only compare images with the same PSF during the clutter filtering process, we must separate our slow-time signal into polyphase channels with the same steering angle and process them independently. This separation results in *N_T_* different polyphase channels operating at a slow-time sampling rate equivalent to that of the HRIs. Using the principles of multirate signal processing, one can show that compounding the LRIs after filtering this way will result in the same digital frequency response as a simple clutter filter on the HRIs (see Theorem A2 in the Appendix). This result is a direct consequence of one of the noble identities of multirate signal processing [14].

It is important to note that using such a filtering scheme comes with some significant drawbacks. Because the clutter filtering occurs at the HRI frame rate, the frequency response of the filter repeats *N_T_* times when analyzed with respect to the LRI frame rate. This results in stop bands occurring at integer multiples of the HRI frame rate. Therefore, velocity estimates within these slow-time bands will be less consistent than at other frequencies.

#### 2.1.5. Lag-One Estimation

In order to estimate the axial motion at each pixel in the image, a lag-one autocorrelation approach was used [2]. Using the beamformed, complex analytic signal *s_a_*(*~r*, *t*) at each pixel location, the complex slow-time autocorrelation was evaluated at a single sample lag via conjugate product followed by convolution with a rectangular filter, yielding

(4) $$R_{1}(\vec{r},t,\tau =\Delta t)=\text{rect}\left(\frac{t}{T}\right)\underset{t}{*}\left(s_{a}^{*}(\vec{r},t)s_{a}(\vec{r},t+\Delta t)\right),$$

where $R_{1}(\vec{r},t,\tau )$ is the autocorrelation estimate at location $\vec{r}$, *t* is the slow-time instant, *τ* is the lag, *T* is the averaging interval, and Δ*t* is the sampling interval. The instantaneous phase shift is then estimated via an arctangent and converted to velocity via the Doppler equation, yielding

(5) ϕ^(r→,t)=atan2(Im{R1(r→,t,Δt)},Re{R1(r→,t,Δt)}

(6) $$\hat{\omega }\left(\vec{r},t\right)=\frac{\hat{\phi }\left(\vec{r},t\right)}{\Delta t}$$

(7) $${\hat{\nu }}_{z}\left(\vec{r},t\right)=\frac{\hat{\omega }\left(\vec{r},t\right)}{2\pi f_{0}}\frac{c}{2}$$

where $\hat{\phi }$ is the phase estimate in radians, $\hat{\omega }$ is the instantaneous slow-time frequency estimate, ${\hat{\nu }}_{z}$ is the velocity estimate, *f*_0_ is the transmitted center frequency, and *c* is the sound speed of the medium. Due to the phase ambiguity of the arctangent function, possible values for $\hat{\phi }$ are limited to the interval (−*π*, *π*]. Because of this ambiguity, the phase-based velocity estimation algorithms exhibit aliasing when the velocity magnitude exceeds the slow-time Nyquist rate, given by

(8) $$\nu_{N}\equiv \max{\hat{\nu }}_{z}=\frac{f_{N}}{f_{0}}\frac{c}{2}$$

(9) $$=\frac{1}{2\Delta tf_{0}}\;\frac{c}{2}$$

where *f_N_* = 1/(2Δ*t*) is the Nyquist frequency. For this reason, it is advantageous to utilize the smallest sampling interval possible when estimating high velocities using high-frequency ultrasound.

#### 2.1.6. Pairwise Lag-One Estimation

The lag-one estimation algorithm described above is versatile enough to be used with either compounded or precompounded images, with the latter being advantageous from a Nyquist standpoint, as it allows for a shorter sampling interval. However, using the lag-one estimation algorithm on the LRIs may result in artifacts due to the rotation of the PSF. By repeating each angle immediately after transmission, we can eliminate these artifacts while simultaneously maintaining the short slow-time sampling interval characteristic of the LRIs. This leads to a variant of the lag-one estimation algorithm which we shall call pairwise lag-one estimation. In pairwise lag-one estimation, we form the short-time autocorrelation estimates only on adjacent LRI pairs which share the same transmission angle (and therefore the same PSF). For notational purposes, let us define the slow-time complex analytic signal corresponding to angle *ϕ_i_*, repeat channel *m*, and slow-time sample *n* as

(10) $$s_{a}(\vec{r},m,\phi_{i},n)=s_{a}(\vec{r},t(m,i,n))$$

where

(11) $$t(m,i,n)=(nN_{T}+2i+m)\Delta t$$

(12) $$i\in \{0:N_{\phi }-1\}$$

(13) m∈{0:1}

(14) n∈ℤ

(15) $$\phi_{i}=i\Delta \phi +\phi_{0}$$

(16) $$N_{T}=2N_{\phi }$$

and *N_ϕ_* is the number of angles used in the transmission sequence.

Using this convention, we may now define a slow-time lag-one autocorrelation estimate for each angle via conjugate product such that

(17) $${\hat{R}}_{2}(\vec{r},t=(2i+\mathrm{nNT})\Delta t,\tau =\Delta t)=s_{a}^{*}(\vec{r},0,\phi_{i},n)s_{a}(\vec{r},1,\phi_{i},n).$$

By defining the autocorrelation this way, adjacent autocorrelation estimates are now temporally indexed at half the LRI sampling rate, but are still lagged by one LRI slow-time sample. This effectively allows for phase estimation at the LRI frame rate, but without any motion artifacts due to the rotation of the PSF. At this point, averaging may be performed at this half sample rate via a convolution with a rectangular filter yielding

(18) $$R_{2}(\vec{r},t,\Delta t)=\text{rect}{\left(\frac{t}{T}\right)}_{\overset{*}{t}}{\hat{R}}_{2}(\vec{r},t,\Delta t).$$

From this point, the velocity estimation process is the same as given in Equations (5)–(7).

#### 2.1.7. HRI Pairwise Lag-One Estimation

Yet another approach that can be pursued with this estimation scheme is using an interleaved compounding scheme. In this case, we generate two HRI channels corresponding to the value of *m*, given by

(19) $$h_{a}(\vec{r},m,n)=\sum_{i=0}^{N_{\phi }-1}s_{a}(\vec{r},m,\phi_{i},n).$$

In the case depicted in Figure 1b, the coherent compounding produces two HRIs per transmit sequence period, one each for the blue and green subsets. Each compounded frame is thus made up of samples spaced at an interval of 2Δ*t*, but each component of these HRI channels has a lag of Δ*t* relative to its partner. This way, we can boost the signal for slowly moving points in the field, but still have relative lags at the LRI time scale. The lag-one autocorrelation estimate is then given by

(20) $${\hat{R}}_{3}(\vec{r},t=nN_{T}\Delta t,\tau =\Delta t)=h_{a}^{*}(\vec{r},0,n)h_{a}(\vec{r},1,n)$$

(21) $${\hat{R}}_{3}(\vec{r},t=nN_{T}\Delta t,\tau =\Delta t)=\text{rect}{\left(\frac{t}{T}\right)}_{\overset{*}{t}}{\hat{R}}_{3}(\vec{r},t,\Delta t).$$

In this case the autocorrelation is sampled in intervals of *N_T_*Δ*t* but again the lag is estimated at an interval of Δ*t*, allowing for a higher Nyquist velocity. From this point, the velocity estimation process is the same as given in Equations (5)–(7).

### 2.2. Experimental Design

#### 2.2.1. Ultrasound Acquisition

Data were acquired with a Vantage 128 ultrasound research platform (Verasonics, Redmond, WA) and an 15.6-MHz, 128-element linear array (Verasonics L22-14v) (Table 1). Plane waves were transmitted at a PRF of 30 kHz using a Tukey cosine apodization with a 10% rolloff parameter. The transmit frequency was 17.857 MHz, the duration of the transmit waveform was 1.5 cycles, and the transmission angles ranged from −**5 to 5 degrees. Received data were bandpass filtered by the analog frontend at a center frequency of 15.625 MHz and sampled at 4 samples per wavelength at this new center frequency. This frequency was chosen due to a maximum sampling frequency constraint of 62.5 MHz on the Vantage’s analog to digital converter.

A real-time display mode was used to select an imaging plane, and then high-speed plane-wave acquisition was initiated. After all of the transmissions, pre-beamformed channel data were streamed to the host PC and the system was returned to the real-time mode. Beamformed in-phase (I) and quadrature (Q) signal components were generated using a graphic-processing-unit (GPU) based algorithm with an unsteered, 128-element Hann-apodized receive aperture [16].

#### 2.2.2. Rotation Phantom

**Figure 3:**
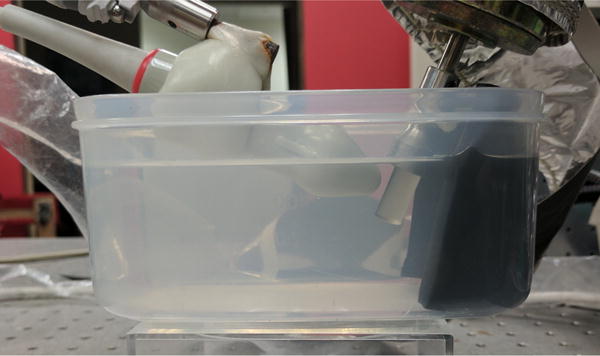
Experimental Setup. A linear 128-element linear array transducer was submerged in a degassed water bath and aligned with a hyperechoic rotation phantom. A layer of sound absorbing material (Sorbothane Inc., Kent, OH, USA) was used to suppress reverberation echoes from the edge of the plastic container.

In order to assess the performance of the transmit sequences, an experiment was conducted using a rotating cylindrical hyperechoic phantom (ATS Laboratories, Bridgeport, CT, USA). The 10-mm diameter phantom was mounted onto a motor shaft and aligned with the linear array transducer (Figure 3). The phantom and transducer were submerged in a degassed water bath and a piece of sound absorbing material (Sorbothane Inc., Kent, OH, USA) was placed behind the phantom to suppress reverberation artifacts. The rotation phantom was spun at a constant rate of 12 revolutions per second. Data were acquired for two full revolutions of the phantom and beamformed. A position-based external trigger was used to ensure that all acquired data would be temporally synchronized.

#### 2.2.3. Transmission Sequences

Two of the transmission sequences were standard linear three-angle single transmit sequences (sT×3, {−5, 0, 5}) or seven-angle single transmit sequences (sT×7, {−5 : 5/3 : 5}). third sequence was a double transmit sequence using 3 angles and, therefore, was composed of 6 transmits (dT×6, {−5, −**5, 0, 0, 5, 5}). In each case, an odd number of angles was chosen to include the 0-degree steered transmit, which is least susceptible to grating lobes. Under this constraint, the sT×7 dataset was chosen to be a partial control for the sequence duration.

**Figure 4:**
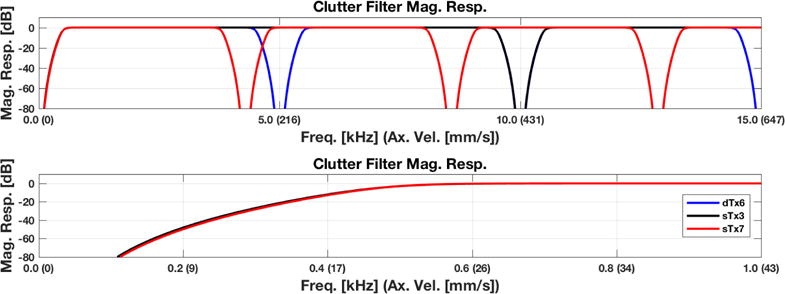
Clutter filter magnitude response. One should note the induced periodicity of the stop bands. This periodicity is due to the design decision to filter the LRI polyphase channels independently, as explained in Section 2.1.4. The repeated notch locations of the filter are located at integer multiples of 2*N_T_*/*f_N_*. These notch locations correspond to the frame rates of HRIs, and consequentially the individual LRI polyphase channel frame rates as well. A zoomed in version is displayed below to help visualize stop band performance.

Each dataset was processed using the LRIs and HRIs using the lag-one estimator described in Section 2.1.5. For the HRIs, a standard sixth order infinite impulse response filter (IIR) Butterworth filter was used for clutter filtering, with a slow-time cutoff frequency of 500 Hz (≈24.6 mm/s). The cutoff frequency of this filter was chosen to compensate for the slow transition band of the Butterworth filter, which takes approximately 300 Hz to reach a 60-dB attenuation level. For the LRIs, clutter filtering was performed on the matched angle LRI channels as described in Section 2.1.4, with similarly designed specifications at the appropriately reduced sample rates as shown in Figure 4. This design decision resulted in a periodic notching effect, in accordance with multirate signal processing theory [14,15].

The expected Nyquist velocities for each of these approaches are given in Table 2, in addition to other relevant slow-time quantities. In addition to the traditional approaches described above, the dTx6 dataset also was post processed using the pairwise (PW) methods described in Sections 2.1.6 (dTx6 LRI PW) and 2.1.7 (dTx6 HRI PW). The baseline averaging interval for each processing scheme was chosen to be around 1 ms in each case. Because the frame rate of the autocorrelation sequence varied so much across all the different processing schemes, matching the averaging schemes exactly was not possible. Furthermore, to avoid introducing half sample lags, odd length averages were used to keep the sequences temporally synchronized. These decisions resulted in the slight variations in the averaging interval as shown in Table 2.

In addition to the color flow Doppler images, power Doppler images were also generated to help visualize the spatial energy distributions. These images were generated by calculating the mean-square value of the slow-time signal over the entire slow-time dimension, which was then converted to a decibel scale, such that

(22) $$P\left(\vec{r}\right)=10\;\log_{10}\left(\frac{1}{N}\sum_{n=0}^{N-1}s{\left(\vec{r},n\Delta t\right)}^{2}\right),$$

where $P\left(\vec{r}\right)$ is the power Doppler signal at the location $\vec{r}$, *N* is the total number of slow-time samples, and $s\left(\vec{r},t\right)$ is the slow-time signal for either the LRIs or HRIs as appropriate. Note that in the LRI case, we do not evaluate the power Doppler on the individual polyphase channels, but rather the full slow-time signal. For the pairwise estimation schemes, no power Doppler images were produced due to lack of a relevant metric which accounts for the pairwise nature of the signals. In practice, the LRI power Doppler could serve as a relevant metric for the pairwise approaches for the purposes of stationary clutter suppression.

As a ground truth for comparison purposes, a rotation model was fit to the data. Using a B-Mode image as a reference, points along the phantom boundaries were manually selected, and the centroid was computed. A 10-mm circular mask was then centered at the estimated centroid, and the axial component of the velocity was modeled using

(23) $$v_{z}\left(x\right)=\omega_{\text{Phantom}}\times \left(x-\bar{x}\right)$$

where *ω*_Phantom_ is the angular velocity of the phantom and $\bar{x}$ is the *x*-component of the centroid. Using this ground truth model, the root-mean-square (RMSE) and bias errors were quantified. Because the phantom was also prone to precession, the standard deviation was also computed as a reference metric to account for any precessional and alignment biases. To facilitate easy comparison between all the methods, each metric was averaged over slow-time and depth so that they could be overlaid on the same plot for comparison purposes.

## 3. Results

**Figure A1:**
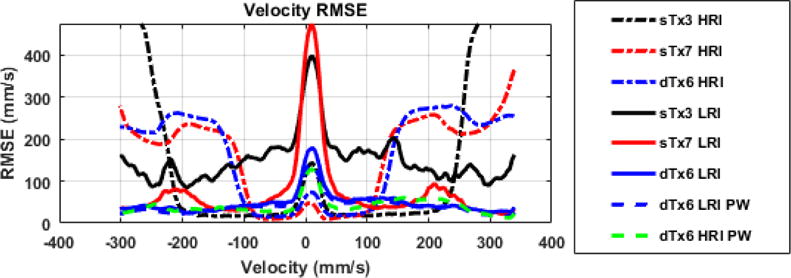
In the HRI estimators, the aliasing manifests as plateauing in the root-mean-square error (RMSE), resulting from the lower frame rate. All other estimators appear to predominately track the standard deviation shown in Figure A2.

**Figure A2:**
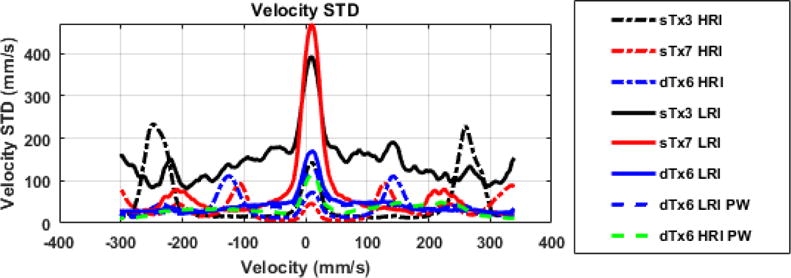
For the traditional HRI estimators, error resonates at the Nyquist velocities, likely due to the aliasing. The sTx3 LRI estimator appears to have uncharacteristically high error across the velocity range, suggesting that the individual LRIs are highly decorrelated, possibly due to a high angular step. In the sTx7 LRI dataset, the error resonates at the velocity associated with the transmit sequence period, likely due a combination of sidelobe motion and attenuation due to the clutter filter. The dTx6 estimators do not have a corresponding peak, suggesting an inherent robustness to these effects.

**Figure A3:**
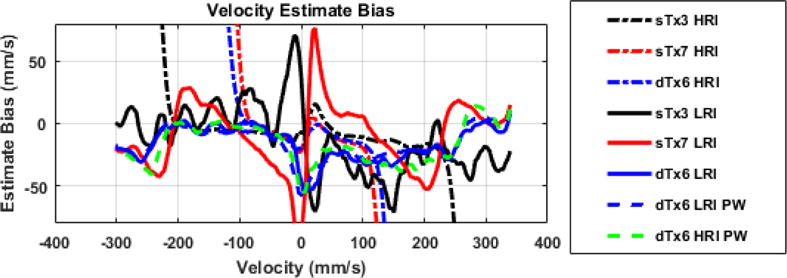
All estimators exhibit a slight negatively sloping trend, suggesting a possible alignment error in the ground truth. The HRI estimators each show evidence of aliasing at the predicted Nyquist velocities.

The results of the rotation phantom experiment are shown in Figures A1–A3. As can be observed in Figure A1, all the HRI estimates exhibit some degree of aliasing, as evidenced by the large errors at high velocities, with the three-angle single transmit sequence (sTx3 HRI) exhibiting the widest usable velocity range of all processing schemes using traditional processing. This result is predicted by the traditional Doppler slow-time frequency analysis, because with only three transmit events, the compounded dataset has the highest slow-time frame rate of the group, and thus the highest velocity range. Note that in each case, the aliasing error begins at lower velocities than predicted in Table 2. This is due to the broadband nature of the excitation. Because the excitation possesses frequency content above the center frequency, aliasing begins before the prediction, because the high-frequency components alias before the center frequency reaches its Nyquist rate. More conservative estimates could have been done by considering the entire band of the excitation, but such estimation schemes deviate from established conventions and were thus avoided to facilitate easy comparison.

Comparing the traditional lag-one estimates of the LRI processing schemes, one can observe that the sTx3 dataset is characterized by large RMSE and standard deviations across the velocity range. This would suggest low correlations in the individual slow-time lines. It is possible that in this case the sidelobe motion might have been fast enough to corrupt the estimate due to the high angular step, even despite the aggressive clutter filtering employed. This could possibly explain why the performance is recovered in the sTx3 HRI scheme.

**Figure A5:**
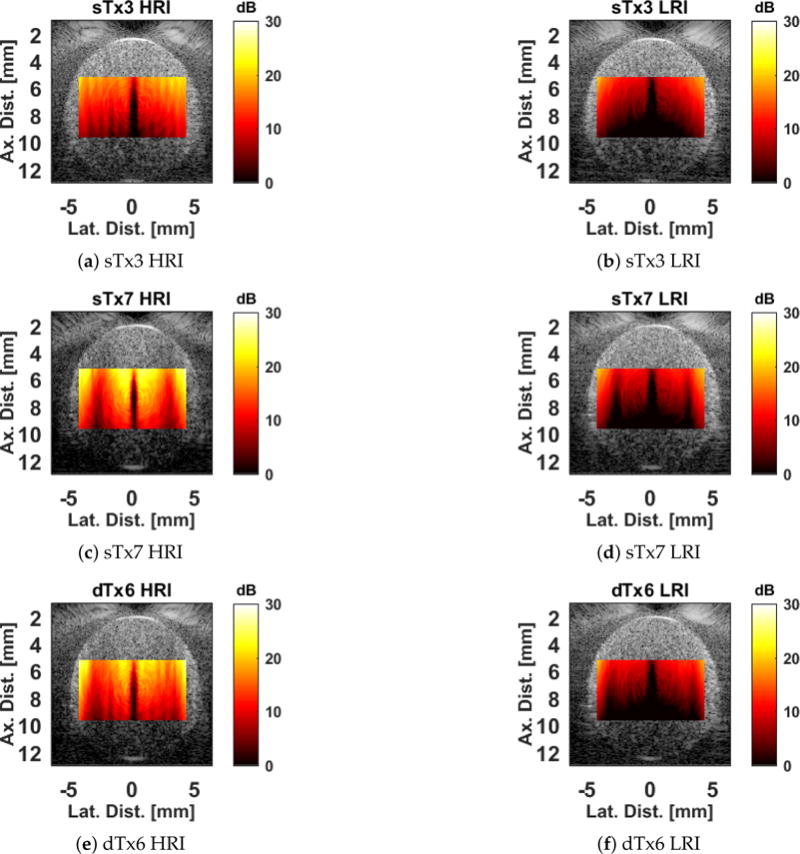
Power Doppler images generated from the experimental data. The HRI images are shown on the left, and the LRI images are shown on the right, with each row corresponding to a different dataset. The pairwise results are omitted, since they lacked a descriptive metric to characterize the power in such a way that would account for the pairwise estimation scheme. One should note the presence of spatial notches in the images. These notches correspond to velocities in the clutter filter stop band. The notches appear to spread out with depth, which is believed to be due the cumulative effect of attenuation. All images are rendered with the same color scale to facilitate easy comparisons. The lateral banding the sTx3 and dTx6 HRI processing schemes is believed to be due to an artifact in the broadside transmission that is specific to the Verasonics Vantage system. This artifact is likely due to the simultaneous excitation of all the elements. The effect is suppressed in the sTx7 dataset due to the additional steered excitations which do not exhibit this effect.

For the seven-angle dataset (sTx7), the slow-time signal error resonates at around 200 mm/s. This error is anticipated due to the clutter filter stop band at that particular velocity range (see Figure 4). The effect also manifests in the power Doppler image in this dataset (Figure A5), because the signal is attenuated in the corresponding spatial region. This error is also exacerbated due to the sidelobe motion being present at those frequencies. While one could argue that such estimates would be suppressed by power Doppler thresholding, the fact remains that there is a weak spot in the velocity range that cannot be overcome using a conventional transmit sequence. Similarly, the velocity estimate is also extremely poor near the stationary regime, but because the signal-to-noise ratio (SNR) is low here, this is to be expected.

This secondary error resonance at the compounded frame rate is not present using the traditional double-transmit method (dTx6). Even though the signal power is lower at these velocities, the fact that the sidelobes move at a slower rate allows for a more robust estimate across a larger range than the sTx7 dataset, despite having comparable channel frame rates. The error in the near stationary regime is also markedly better than both the sTx LRI processing schemes. Both pairwise estimation schemes had only minor improvements in the dTx dataset, suggesting that the dominant improvement is the temporal non-uniformity of the sidelobe motion. The interleaved compounded dataset (dTx6 HRI PW), offered a compromise between full compounding (dTx6 HRI) and no compounding (dTx6 LRI), as it tracks well in low velocity regime, but not quite as well in the higher velocity regime.

## 4. Conclusions

In this study, we investigated the effects of using a double transmit sequence to simultaneously expand the Nyquist velocity range in such a way that it is resistant to sidelobe motion. Use of the double transmit sequence allowed for significant performance improvements in velocity regimes that would typically be attenuated by clutter filtering, demonstrating increased robustness to noise. The proposed methods significantly outperform the traditional methods in the high velocity regime in both root-mean square-error and standard deviation, with minimal performance losses in the low-velocity regime. These results suggest a new paradigm in velocity estimation in the context of high-frame-rate Doppler ultrasound.

## Figures and Tables

**Table 1:** The relevant transducer specifications and system settings are given below.

Parameter	Value
Excitation Frequency	17.857 MHz
Received Center Frequency	15.625 MHz
Received Center Wavelength	98.56 μm
Received Bandwidth	14–22 MHz
Elevation Focus	8 mm
Element Height	1.5 mm
Element Width	80 μm
Element Pitch	100 μm/1.01*λ*

**Table 2:** The relevant slow-time quantities are given below. Note that in the case of the pairwise (PW) estimates in the last two rows the correlation frame rate differs from the in-phase and quadrature (IQ) frame rate. This is a characteristic of the pairwise estimation schemes. sTx3: three-angle single transmit sequence; sTx7: seven-angle single transmit sequence. dTx6: three-angle, double transmit sequence.

Estimate	IQ Frame Rate (kHz)	Corr. Lag (μs)	Nyquist Velocity (mm/s)	Corr. Frame Rate (kHz)	Averaging Interval (Samples)	Averaging Interval (ms)
sTx3 HRI	10	100.0	246.4	10	11	1.100
sTx7 HRI	4.286	233.3	105.6	4.286	5	1.167
dTx6 HRI	5	200.0	123.2	5	5	1.000
sTx3 LRI	30	33.3	739.2	30	31	1.033
sTx7 LRI	30	33.3	739.2	30	31	1.033
dTx6 LRI	30	33.3	739.2	30	31	1.033
dTx6 LRI PW	30	33.3	739.2	15	15	1.000
dTx6 HRI PW	30	33.3	739.2	5	5	1.000

**Figure A4:**
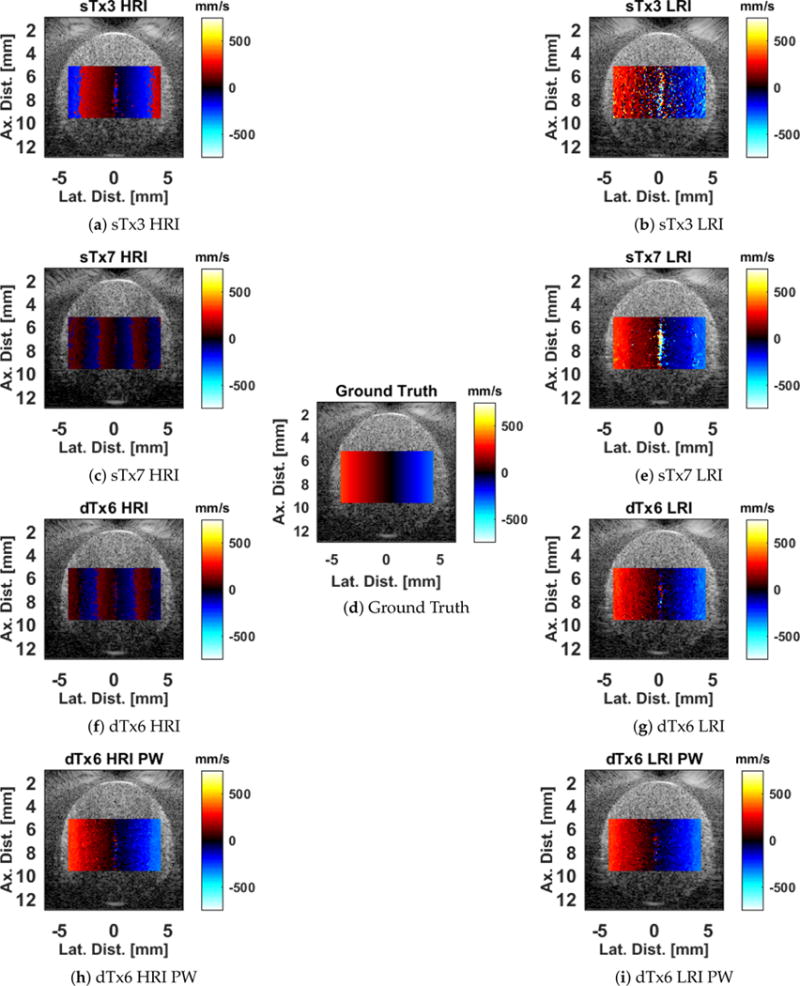
Color flow Doppler images generated from the experimental data. The HRI images are shown on the left, and the LRI images are shown on the right, with each row corresponding to a different dataset. The pairwise results are shown in the last row. All images are rendered with the same color scale to facilitate easy comparisons. Aliasing is clearly observed in the traditional HRI cases, manifesting as banding artifacts. The high error observed in the center is due to the clutter filter, which attenuates slow-moving components of the signal. This confirmed in the power Doppler images (Figure A5), which exhibit deep notches in the center of the image, corresponding to stationary axial motion.

## Appendix A. Multirate Theorems of Coherent Plane Wave Compounding

Let $s[n]\equiv s_{a}(\vec{r},n\Delta t)$ be the discrete, slow-time, precompounded, analytic signal at a location $\vec{r}$. For compactness of notation, the position argument $\vec{r}$ is suppressed in the following section, with the understanding that it is evaluated independently at every spatial location.

### Theorem A1

The coherent plane wave compounded signal *s*_+_[*n*] is equivalent to a rectangular filtering of *s*[*n*] followed by a downsampling operation.

#### Proof

By definition, *s*_+_[*n*] is given by

(A1) $$s_{+}[n]≜\sum_{n^{\prime }=0}^{N_{T}-1}s[N_{T}n+n^{\prime }].$$

Let us define the rectangular signal that is nonzero on the closed interval [*a*, *b*] as

(A2) $$r_{\left[a,b\right]}[n]\equiv \left\{ \begin{array}{ll} 1, & n\in [a,b]\\ 0, & \text{otherwise}. \end{array}\right.$$

A simple variable substitution of *n′* → −*n′* results in

(A3) $$s_{+}[n]=\sum_{n^{\prime }=-(N_{T}-1)}^{0}s[N_{T}n-n^{\prime }]$$

(A4) $$=\sum_{n^{\prime }=(1-N_{T})}^{0}s[N_{T}n-n^{\prime }]$$

(A5) $$=\sum_{n^{\prime }=-\infty }^{\infty }r_{\left[1-N_{T},0\right]}[n^{\prime }]s[N_{T}n-n^{\prime }]$$

(A6) $$=s_{f}[N_{T}n],$$

where *s_f_* [*n*] is the rectangularly filtered analytic signal given by

(A7) $$s_{f}[n]=r_{\left[1-N_{T},0\right]}{\left[n\right]}_{\overset{*}{n}}s[n]$$

(A8) $$=\sum_{n^{\prime }=-\infty }^{\infty }r_{\left[1-N_{T},0\right]}[n^{\prime }]s[n-n^{\prime }].$$

Because the downsampling operation by a factor of *N_T_* is defined as

(A9) $$D_{N_{T}}\left\{s_{f}[n]\right\}=s_{f}[N_{T}n]$$

we find that

(A10) $$s_{+}[n]=D_{N_{T}}\{s_{f}[n]\}$$

(A11) $$=D_{N_{T}}\left\{r_{\left[1-N_{T},0\right]}{\left[n\right]}_{\overset{*}{n}}s[n]\right\}.$$

Thus, the coherent compounding operation is equivalent to a rectangular filter of length *N_T_* followed by a downsampling of *N_T_*.□

### Theorem A2

Consider the traditional pipeline of passing the compounded signal *s*_+_[*n*] through a high-pass clutter filter with impulse response *h*[*n*]. Then, the output signal *s_c_*[*n*] is equivalent to filtering each individual polyphase channel $s_{\phi_{n^{\prime }}}[n]$ and summing.

#### Proof

Let us denote the output of the clutter filter as *s_c_*[*n*], such that

(A12) $$s_{c}[n]=h{\left[n\right]}_{\overset{*}{n}}s_{+}[n].$$

Thus, we have

(A13) $$s_{c}[n]=h{\left[n\right]}_{\overset{*}{n}}\left(\sum_{n^{\prime }=0}^{N_{T}-1}s[N_{T}n+n^{\prime }]\right)$$

Let us define the polyphase channel $s_{\phi_{n^{\prime }}}[n]$ as the slow-time signal associated with the *n′*th transmit in the transmit sequence period. Mathematically the relationship is described by

(A14) $$s_{\phi_{n^{\prime }}}[n]\equiv s[N_{T}n+n^{\prime }].$$

Thus, due to the commutativity of convolution and summation, we have

(A15) $$s_{c}[n]=h{\left[n\right]}_{\overset{*}{n}}\left(\sum_{n^{\prime }=0}^{N_{T}-1}s_{\phi_{n^{\prime }}}[n]\right)$$

(A16) $$=\sum_{n^{\prime }=0}^{N_{T}-1}\left(h{\left[n\right]}_{\overset{*}{n}}s_{\phi_{n^{\prime }}}[n]\right).$$

Therefore, filtering each individual polyphase component before summation yields the same response as filtering the compounded signal. □

## Abbreviations

**The following abbreviations are used in this manuscript**
PRF: Pulse repetition frequency
HRI: High-resolution image/compounded image
LRI: Low-resolution image/pre-compounded image
PSF: Point spread function
RMSE: Root-mean-square error
sTx: Single transmit sequence (dataset prefix)
dTx: Double transmit sequence (dataset prefix)
PW: Pairwise, (estimation scheme suffix)
SNR: Signal-to-noise ratio
